# Deep learning-enabled fast DNA-PAINT imaging in cells

**DOI:** 10.52601/bpr.2023.230014

**Published:** 2023-08-31

**Authors:** Min Zhu, Luhao Zhang, Luhong Jin, Yunyue Chen, Haixu Yang, Baohua Ji, Yingke Xu

**Affiliations:** 1 Department of Biomedical Engineering, Key Laboratory of Biomedical Engineering of Ministry of Education, State Key Laboratory of Extreme Photonics and Instrumentation, Zhejiang Provincial Key Laboratory of Cardio-Cerebral Vascular Detection Technology and Medicinal Effectiveness Appraisal, Zhejiang Provincial Key Laboratory of Traditional Chinese Medicine for Clinical Evaluation and Translational Research, Zhejiang University, Hangzhou 310027, China; 2 Binjiang Institute of Zhejiang University, Hangzhou 310053, China; 3 Department of Engineering Mechanics, Biomechanics and Biomaterials Laboratory, Zhejiang University, Hangzhou 310027, China; 4 Department of Endocrinology, Children’s Hospital of Zhejiang University School of Medicine, National Clinical Research Center for Children’s Health, Hangzhou 310051, China

**Keywords:** DNA-PAINT, U-Net, SMLM, Super-resolution imaging, Deep learning

## Abstract

DNA-based point accumulation in nanoscale topography (DNA-PAINT) is a well-established technique for single-molecule localization microscopy (SMLM), enabling resolution of up to a few nanometers. Traditionally, DNA-PAINT involves the utilization of tens of thousands of single-molecule fluorescent images to generate a single super-resolution image. This process can be time-consuming, which makes it unfeasible for many researchers. Here, we propose a simplified DNA-PAINT labeling method and a deep learning-enabled fast DNA-PAINT imaging strategy for subcellular structures, such as microtubules. By employing our method, super-resolution reconstruction can be achieved with only one-tenth of the raw data previously needed, along with the option of acquiring the widefield image. As a result, DNA-PAINT imaging is significantly accelerated, making it more accessible to a wider range of biological researchers.

## INTRODUCTION

Super-resolution microscopy has emerged as a promising tool in recent years, enabling researchers to study cellular structures and biological processes at the molecular scale. Typically, super-resolution imaging strategies can be categorized into two groups (Schnitzbauer *et al.* 2017). The first group is illumination-pattern-based methods, such as structured illumination microscopy (SIM) (Chen *et al.* 2022; Gustafsson 2005) and stimulated emission depletion (STED) microscopy (Alvelid *et al.* 2022; Hell *et al.* 1994). The first group has a broader range of fluorophore selection, but requires more complex instrumental setups. The second group consists of single-molecule-localization-based methods, such as photo-activated localization microscopy (PALM) (Betzig *et al.* 2006; Xie *et al.* 2020) and stochastic optical reconstruction microscopy (STORM) (Rust *et al.* 2006; Xu *et al.* 2020), which achieve sub-diffraction precision by exploiting stochastic blinking of specific fluorescent probes.

In particular, single-molecule localization microscopy (SMLM) has been widely employed to achieve a ten-fold increase in resolution by sequentially exciting and detecting fluorophores and reconstructing their position in nanometric precision. SMLM has enabled the imaging of subcellular structures beyond the diffraction limit. SMLM can be further divided into three main families depending on the mechanism of single-molecule fluorescent signal separation (Tholen *et al.* 2023): (1) photo-activation/switching based microscopy, including PALM, STORM; (2) dynamic labelling and reversible binding based microscopy exemplified by point accumulation for imaging in nanoscale topography (PAINT) (Giannone *et al.* 2010; Sharonov *et al.* 2006); and (3) fluorescence lifetime separation based microscopy (Oleksiievets *et al.* 2022). Among them, PAINT stands out due to straightforward implementation that does not require special experimental conditions for photoswitching, as long as probes possess the capability to diffuse and reach their target structures. The concept of PAINT relies on the premise that fluorescent probes, targeting specific structures, freely diffuse within the solution. However, as interactions are mainly limited to hydrophobic interactions or electrostatic coupling, the original implementation of PAINT poses challenges in selectively labelling a diverse range of biomolecules.

**Figure 1:**
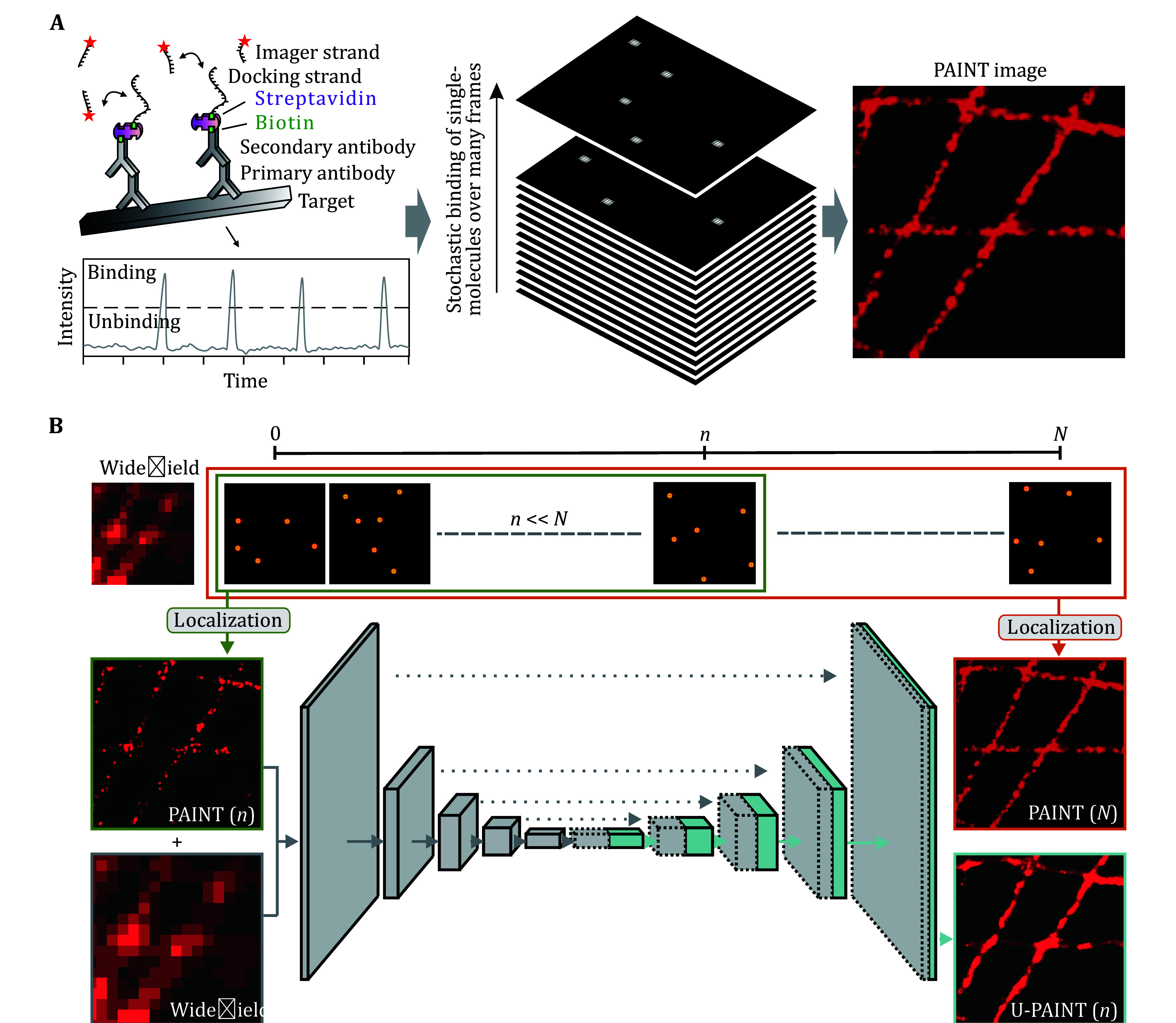
A brief overview of the deep learning-enabled fast DNA-PAINT. **A** Principles of traditional DNA-PAINT. The target structure is sequentially connected to the primary antibody, biotin-labeled secondary antibody, Alexa Fluor 488 conjugated streptavidin, and biotin-labeled DNA docking strand. Cy3B labeled DNA imaging strand is introduced in the imaging buffer. Both docking strands and imaging strands are single-stranded DNA with partially complementary sequences. Accumulation of single-molecule fluorescence signals left by stochastic binding of imager strands generates a comprehensive representation of structural information. **B** Principles of fast DNA-PAINT with U-Net. Traditional DNA-PAINT utilizes tens of thousands of frames (*N*) of raw data to reconstruct one super-resolution image. When raw data is insufficient (*n*, *n* << *N*) for traditional DNA-PAINT reconstruction, our deep learning-based approach can still reconstruct comprehensive super-resolution images

DNA nanotechnology offers a promising approach to leverage the benefits of the PAINT concept while establishing a programmable target-probe interaction system. DNA-based PAINT (DNA-PAINT) has been developed as a straightforward approach to overcome some limitations of current localization-based super-resolution techniques (Dai *et al.* 2016; Iinuma *et al.* 2014; Jungmann *et al.* 2010, 2014, 2016). DNA-PAINT decouples blinking from the photophysics of dye and introduces the programmability and specificity of using DNA molecules as imaging and labeling probes. A DNA-PAINT system comprises a docking strand and a complementary imager strand, both of which are short single-stranded DNA oligomers, usually 8–10 nucleotides long. The docking strand is attached to the target of interest using immunolabeling approaches with DNA-conjugated antibody pair targeting proteins of interest. The imager strand is conjugated to an organic dye and diffuses freely in the imaging buffer (Fig. 1A). Generally, unbound imager strand signals appear imperceptible in the camera because they diffuse across multiple camera pixels during the exposure time. However, imager strands can transiently bind to docking strands owing to their complementary sequence. In a bound state, when imager strands remain anchored in place for an extended period, accumulating sufficient photons to be detected by a single camera pixel. The properties of DNA-PAINT offer several advantages over other SMLM approaches. Firstly, the use of DNA-based imaging probes enables high multiplexing using Exchange-PAINT limited only by the number of orthogonal DNA sequences, in contrast to the spectrally distinct dyes used in conventional multiplexing experiments (Jungmann *et al.* 2014). Secondly, the predictability and tunability of DNA binding and unbinding events, coupled with the absence of bleaching, allow for accurate quantitative image interpretation, as implemented in quantitative PAINT (qPAINT) (Jungmann *et al.* 2016). Thirdly, DNA-PAINT simplifies the selection of suitable dyes for imaging, as the parameter space is reduced from rather complex photophysical properties to basically one single parameter — the photon budget. Lastly, by programming the binding duration, a significantly higher number of photons can be detected from a single binding (or blink) event, leading to optimal localization precision.

Although DNA-PAINT offers several advantages over traditional super-resolution techniques, it is important to acknowledge the current limitations. Firstly, DNA-PAINT is currently limited to fixed specimens. Live-cell imaging could be more challenging compared to the aforementioned techniques. This difficulty arises from the complexities associated with introducing dye-labeled nucleic acid strands into living cells and the potential unforeseen consequences associated with the introduction of nucleic acids. Secondly, DNA-PAINT is primarily compatible with optical sectioning techniques such as total internal reflection (TIR), oblique illumination or light-sheet microscopy due to elevated background fluorescence originating from unbound imager strands. This limitation restricts the applicability of DNA-PAINT to these specific imaging modalities. Furthermore, in order to diminish background fluorescence, the concentration of the imaging strand is constrained to exceedingly low levels. To acquire adequate single-molecule fluorescence data, it is necessary to invest several hours in obtaining tens of thousands of frames of raw data for DNA-PAINT reconstruction. This can pose challenges in dynamic imaging scenarios where temporal resolution is needed.

Extensive efforts have been devoted to accelerating the imaging speed or optimizing imaging conditions for PAINT from the physical and chemical parts of the probe development. Through optimization of DNA hybridization conditions, such as rationally designed DNA sequence and buffer salinity, ten times acceleration has been achieved in DNA-PAINT image acquisition (Schueder *et al.* 2019). Ago-PAINT, which is based on protein-assisted delivery of DNA imager strands, has a 10-fold higher binding rate compared to conventional DNA-PAINT and allows for collecting more single molecules in a given time window (Filius *et al.* 2020). Fluorogenic DNA-PAINT, using an imager strand with both fluorophore and quencher, has emancipated DNA-PAINT from reliance on optical sectioning techniques and significantly elevates the contrast (Chung *et al.* 2022). Besides, algorithmic methods would further accelerate PAINT imaging, especially by deep-learning approaches. Thus, here we introduce a U-Net based deep learning method to substantially reduce the demand for raw data in DNA-PAINT imaging. Deep learning, a methodology of machine learning, represents a significant subdivision of artificial intelligence (Marx 2019). Deep learning typically invites the use of multifaceted computational units in artificial neural networks, often referred to as "neurons" due to their resemblance to neural cells in biological neural networks. This technology is designed to gradually unravel complex representations from the given input data, thereby facilitating various data analysis tasks and excelling with superhuman performances in certain delineated realms.

U-Net, a deep convolutional network introduced in 2015, is primarily designed for two-dimensional image segmentation. As depicted in Fig. 1B, the U-Net architecture consists of an encoder (downsampling procedure), a decoder (upsampling procedure) and skip connections, which effectively mitigate information loss during the downsampling process (Ronneberger *et al.* 2015). The U-Net networks are capable of learning distinctive features from various structures, and performing image segmentation accordingly. Additionally, they can utilize these learned features to restore missing structures. By leveraging this capability, it is possible to reconstruct subcellular structures from sparse single-molecule localization data, connecting disjointed structures and forming a comprehensive high-resolution image. Consequently, U-Net networks can serve as substitutes for conventional super-resolution reconstruction, reducing computational complexity (Nehme *et al.* 2018), and diminishing the demand for raw data (Jin *et al.* 2020; Ouyang *et al.* 2018; Zhu *et al.* 2022), and enabling super-resolution reconstruction across imaging systems (Jin *et al.* 2020).

This research aims to establish a simplified DNA-PAINT approach and accelerate the imaging process and reconstruction with the U-Net deep learning strategy. The following detailed procedures would provide a comprehensive overview of DNA-PAINT sample preparation, imaging, and super-resolution image reconstruction steps. Samples are linked to docking strands with antibody pair and biotin-streptavidin interaction; DNA-PAINT data is acquired under TIRF microscopy; super-resolution images are reconstructed with Picasso software and our scripts are developed based on U-Net. Our sampling protocol using biotin-streptavidin interaction is one of the most convenient and cost-effective strategies for DNA labeling of antibodies. The fluorescently labeled streptavidin provides widefield signals of the target structures. By implementing our approach, we significantly reduce the amount of single-molecule fluorescence data required to less than 10% of traditional DNA-PAINT methods, while still achieving comparable spatial resolution. Furthermore, our approach exhibits efficiency across all SMLM imaging techniques and can be easily transplanted to other cellular structures with minimal effort. The utilization of the U-Net deep learning strategy in conjunction with DNA-PAINT not only streamlines the imaging process but also accelerates the reconstruction of super-resolution images. This advancement has the potential to facilitate a wide range of biological research applications and can easily be adapted to study various cellular structures.

## MATERIALS AND EQUIPMENT

### Reagents

• Dulbecco's modified Eagle's medium (DMEM; Cytiva, cat. no. SH30243.01B)

• Penicillin-streptomycin solution (Beyotime, cat. no. C0222)

• Fetal bovine serum (FBS; Sigma-Aldrich, cat. no. F8318)

• Glutaraldehyde (25% aqueous solution) (Sinopharm, cat. no. 30092436)

• Triton X-100 (Sinopharm, cat. no. 30188928)

• Phosphate buffered saline (PBS; Biosharp, cat. no. BL302A)

• NaBH_4_ (Sinopharm, cat. no. 80115816)

• Bovine serum albumin (BSA; Sigma-Aldrich, cat. no. B2064-100G)

• Mouse monoclonal antibody to beta-tubulin (Abcam, cat. no. ab231082)

• Biotin-labeled goat antibody to mouse IgG (Abcam, cat. no. ab6788)

• Alexa Fluor 488 conjugated streptavidin (Invitrogen, cat. no. S32354)

• Docking strands (biotin-TTATACATCTATACATCTA; Sangon Biotech (Shanghai))

• Cy3B conjugated imager strands (TAGATGTAT-Cy3B; Invitrogen)

• MgCl_2_ (Sigma-Aldrich, cat. no. M4880-100G)

• NaCl (Sinopharm, cat. no. 10019318)

• Anhydrous ethanol (Sinopharm, cat. no. 10009259)

### Reagent setup

• Pre-fix buffer**.** Dilute 32 μL Glutaraldehyde (25% aqueous solution), 5 μL Triton X-100 in 1963 μL PBS. This solution can be stored away from light at 4 °C for two weeks. Warm the solution to 37 °C before use.

• Fix buffer**.** Dilute 240 μL Glutaraldehyde (25% aqueous solution), 5 μL Triton X-100 in 1755 μL PBS. This solution can be stored away from light at 4 °C for two weeks.

• NaBH_4_ solution (1 mg/mL)**.** Dissolve the NaBH_4_ in PBS to make the final concentration of 1 mg/mL. This solution can be stored for 20 min at room temperature.

**[CAUTION!]** NaBH_4_ dissolution releases a significant amount of hydrogen gas, and therefore, does not cover its container.

• Blocking buffer**.** Dissolve 0.5 g BSA and 25 μL Triton X-100 in 10 mL PBS. Warm the mixture to 37 °C to facilitate BSA dissolution. Filter the solution with a 0.22 μm polyethersulfone (PES) membrane. This solution can be stored for one month at 4 °C.

• Antibody dilution buffer**.** Mix 3 mL blocking buffer and 2 mL PBS. This solution can be stored for one month at 4 °C.

• Primary antibody solution. Dilute 1 μL mouse monoclonal antibody to beta-tubulin in 999 μL antibody dilution buffer.

• Secondary antibody solution. Dilute 1 μL biotin-labeled goat antibody to mouse IgG in 999 μL antibody dilution buffer.

• Streptavidin solution. Dilute 1 μL Alexa Fluor 488 conjugated streptavidin in 999 μL antibody dilution buffer.

• Docking strand solution. Dissolve docking strands (biotin-TTATACATCTATACATCTA) according to its manual with double-distilled water and get 100× stock solution. Dilute 10 μL docking strand stock solution in 990 μL PBS.

• Imaging buffer. Dissolve imaging strands (TAGATGTAT-Cy3B) according to its manual with double-distilled water and get 100,000× stock solution. Dilute 10 μL 100,000× stock solution in 990 μL double-distilled water and get 1000× stock solution. Dissolve 47.6 mg MgCl_2_ and 29.2 mg NaCl in 10 mL double-distilled water and filter the solution with a 0.22 μm PES membrane. Dilute 1 μL 1000× imaging strand stock solution in 999 μL MgCl_2_ and NaCl solution to make the imaging buffer.

### Equipment

• 35-mm glass bottom cell culture dish (Cellvis, D35-20-1.5-N)

• CO_2_ incubator (Thermo Scientific, 370)

• Orbital shaker (Kylin-Bell Lab Instruments, TS-2)

• 0.22-μm PES filter (Millipore, SLGPR33RB)

• Temperature-controlled bench top centrifuge (Eppendorf, 5804R)

• Centrifuge tubes (1.5 mL, Axygen, NCT-150)

• Centrifuge tubes (15 mL, Crystalgen, 23-2273)

• TIRF microscope (Olympus, IX83)

• Graphic work station with Nvidia GeForce RTX 2080 Ti

## PROCEDURE

### Sample preparation [TIMING ~2 d]

**Figure 2:**
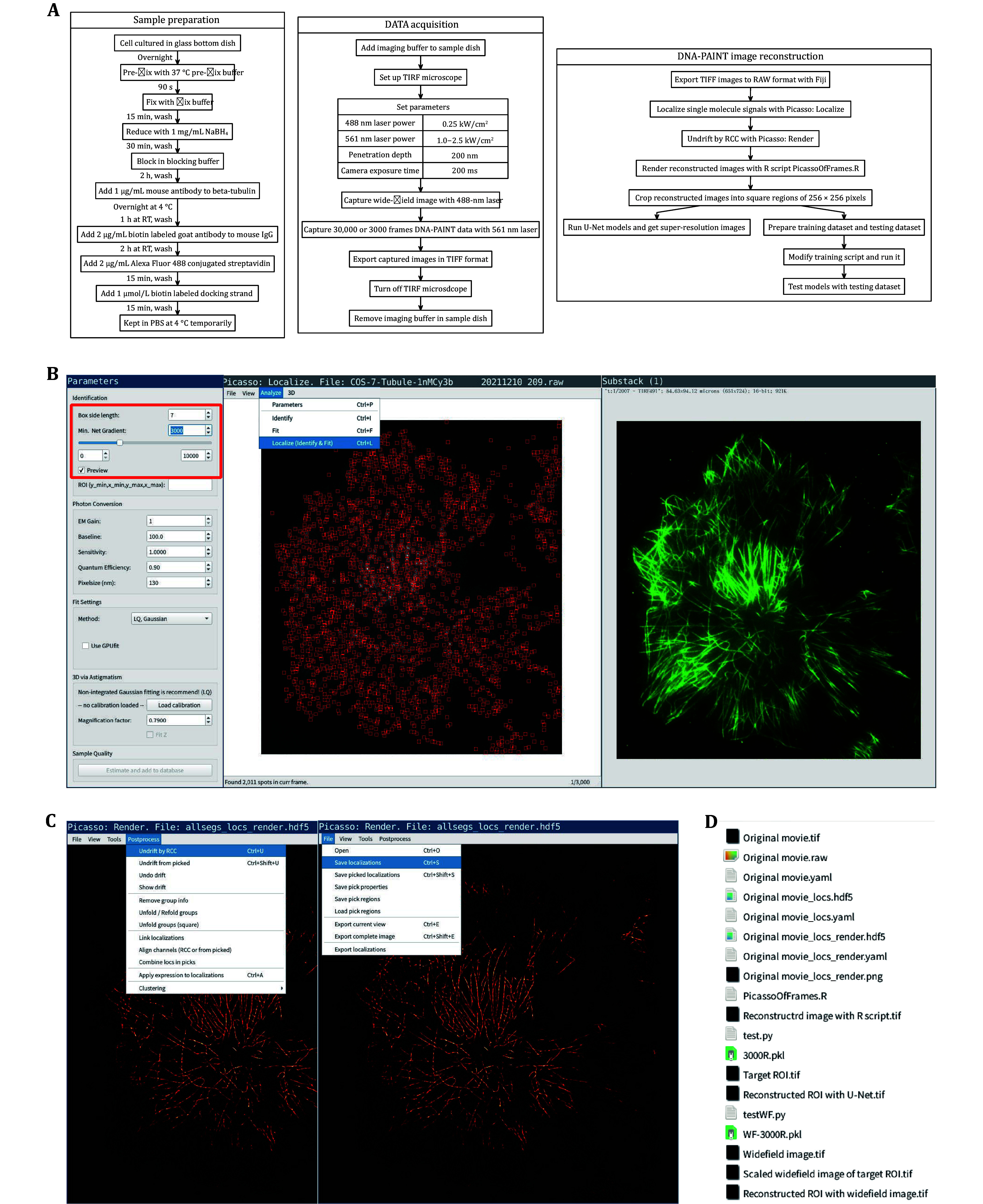
Flow charts and software screenshots of fast DNA-PAINT. **A** Flow charts of fast DNA-PAINT, including sample preparation, TIRF imaging and image reconstruction. **B** Screenshots of “Picasso: Localize” and corresponding widefield image. The optimal range of “Min. net gradient” in the red box varies for each set of DNA-PAINT raw images. **C** Screenshots of “Picasso: Render”. “Picasso: Render” can also perform redundant cross-correlation drift correction. **D** The files used or created during fast DNA-PAINT image reconstruction, including raw images, localization data in HDF5 format, R script and python scripts, U-Net models, and output super-resolution images

Here, we propose a simplified DNA-PAINT labeling method and a deep learning-enabled fast DNA-PAINT imaging strategy for sub-cellular structures, such as microtubules. The complete procedure is presented in Fig. 2A.

1 Total of 150,000–200,000 COS-7 cells are cultured overnight in a 35-mm glass bottom dish containing 2 mL DMEM, supplemented with 1% penicillin-streptomycin and 10% fetal bovine serum at 37 °C in a humidified 5% CO_2_ incubator.

2 Prepare 2 mL pre-warmed pre-fix buffer, and 2 mL fix buffer. Remove DMEM in the cell dish, add pre-fix buffer and incubate for 90 s. Replace pre-fix buffer with fix buffer, and incubate for 15 min.

**[CRITICAL STEP]** Pre-fix buffer should be warmed to 37 °C before use in order to avoid cell shrinking.

3 After fixation, the dish is rinsed three times with 2 mL PBS each time. Prepare 2 mL 1 mg/mL NaBH_4_ in PBS, replace PBS in cell dish and incubate for 30 min.

**[CRITICAL STEP]** To remove chemical ingredients completely, it is recommended to use an orbital shaker during the rinsing step, set the speed to 70–80 r/min, and shake for 5 min each time.

**[CRITICAL STEP]** NaBH_4_ solution must be freshly prepared to reduce NaBH_4_ degeneration in water solution.

4 After NaBH_4_ incubation, the cell dish is rinsed three times with 2 mL PBS each time. Prepare 2 mL of blocking solution, replace PBS in the cell dish with 2 mL blocking buffer and incubate for 2 h.

5 After blocking, the cell dish is rinsed three times with 2 mL PBS each time. Prepare 3.5 mL antibody dilution buffer, and dilute mouse monoclonal antibody to beta-tubulin in 1 mL antibody dilution buffer to 1 μg/mL. Replace PBS in the cell dish with 1 mL primary antibody solution. The cell dish is incubated in a humidified dark box at 4 °C overnight. The rest antibody dilution buffer is kept at 4 °C temporarily.

6 The cell dish in the humidified dark box is incubated at room temperature for 1 h the next day, followed by being rinsed three times with PBS. Dilute-biotin labeled goat antibody to mouse IgG in 1 mL antibody dilution buffer to 2 μg/mL. Add 1 mL secondary antibody solution to the cell dish and incubate for 2 h at room temperature.

7 After biotin-labeled goat antibody incubation, the cell dish is rinsed three times with PBS. Dilute Alexa Fluor 488 conjugated streptavidin to 2 μg/mL with 1 mL antibody dilution buffer. Replace PBS in the cell dish with 1 mL streptavidin solution and incubate for 15 min in the dark.

**[CRITICAL STEP]** The fluorescent dye incubation should be performed in a dark environment.

8 After streptavidin incubation, the cell dish is rinsed three times with PBS. Dissolve biotin-labeled docking strands to 1 μmol/L in PBS. Add 1 mL 1 μmol/L docking strand solution to the cell dish and incubate for 15 min in the dark.

9 The cell dish is rinsed three times with 2 mL PBS each time, and kept in a humidified dark box at 4 °C with 2 mL PBS. The cell dish is now ready for imaging.

### Data acquisition [TIMING ~2 h]

10 Prepare 1 mL imaging buffer. Replace PBS in the sample dish with 1 mL imaging buffer.

11 An Olympus IX83 microscope with a 100× N.A. 1.49 oil objective is used for imaging DNA-PAINT sample. Clean the oil objective and glass bottom dish with lens tissue imbued with anhydrous ethanol. Place the sample on the microscope stage, set the microscope to conventional fluorescence mode, adjust the power density of the 488-nm laser to 0.25 kW/cm^2^, and move the objective up until the sample is in focus. Adjust the stage until the target cell structure is presented in the center of the field of view.

12 Set the TIRF penetration depth to 200 nm, camera exposure time to 200 ms, pixel binning to 2 × 2 pixels, and appropriate wavelength filter. Draw a rectangular ROI precisely covering the target cell structure. Capture a widefield image after fine focusing and export it in TIFF format.

13 Set the power density of the 488-nm laser to 0, the power density of the 561-nm laser to 1.0–2.5 kW/cm^2^, and the appropriate wavelength filter. Capture a movie and export it in TIFF format. For conventional DNA-PAINT imaging, the movie is supposed to contain at least 30,000 frames of single-molecule fluorescence signal data. For U-Net assisted DNA-PAINT, 3000 frames of raw data are required.

14 Clean the oil objective and glass bottom dish with lens tissue imbued with anhydrous ethanol. Replace the imaging buffer in the sample dish with 2 mL PBS and store the sample dish in a humidified dark box at 4 °C. Turn off the microscope.

### Image reconstruction [TIMING ~2 h]

15 Download our scripts and pre-trained models from https://github.com/ccchin999/DNA-PAINT.bpr/releases/latest. Install Nvidia CUDA toolkit, Python (packages listed in “README.md” included), R (packages listed in “README.md” included), Fiji (Schindelin *et al.* 2012) and its plugin raw-yaml exporter according to online manuals. The following procedures are based on an Ubuntu computer with a Nvidia GPU.

16 Launch Fiji and import a single-molecule fluorescence movie file. Run “plugins > raw-yaml Exporter”. A .raw file and a .yaml file are saved in the same path as the movie file.

17 Launch Python software “Picasso: Localize” (Schnitzbauer *et al.* 2017) using terminal command “picasso localize”. Drag .raw file to “Picasso: Localize”. The software interface displaying an opened .raw file is presented on the left side of Fig. 2B, and the corresponding widefield image is shown on the right side of Fig. 2B. Use “View > Contrast” dialog to set image contrast manually to better visualize single molecule signals. Open “Analyze > Parameters” dialog, check “Preview”*,* set “Box side length” to exactly accommodate a single point spread function (PSF), and set proper “Min. net gradient” to distinguish single molecule signals from the background. Inspect corresponding widefield images and ensure no extracellular background signals are identified. Run “Analyze > Localize (Identify & Fit)” to perform the localization process. A locs.hdf5 file and a locs.yaml file are generated automatically in the same path as .raw file.

18 Launch Python software “Picasso: Render” with terminal command “picasso render” and drag locs.hdf5 file to “Picasso: Render”. An 8-bit reconstructed super-resolution image is displayed, as presented in Fig. 2C. Run “Postprocess > Undrift by RCC” for redundant cross-correlation drift correction if drifting is observed during movie acquisition. Run “File > Save localizations” to save corrected localization data in HDF5 format.

19 Run our provided R script using the terminal command: “Rscript /path/to/our/script/PicassoOfFrames.R /path/to/last/hdf5/file /target/path/for/reconstructed/image”. The resulting image is saved in TIFF format.

20 If the obtained single-molecule fluorescence data is insufficient (the image structures are separate points or discontinuous lines), our U-Net model can be used to reconstruct a complete super-resolution image. Crop reconstructed image with insufficient raw data into the square region of 256 × 256 pixels, save them in TIFF format, and run our python script using the terminal command: “python3 /path/to/our/script/test.py /path/to/model/file /path/to/insufficient/tiff/file /path/to/output/tiff/file”. If you choose to add widefield image regions, use “testWF.py” script instead, and run “python3 /path/to/our/script/testWF.py /path/to/model/file /path/to/insufficient/tiff/file /path/to/output/tiff/file /path/to/widefield/file”. If necessary, the outputted 256 × 256 images can be amalgamated for a panoramic view. All files involved in DNA-PAINT image reconstruction are listed in Fig. 2D.

**[CRITICAL STEP]** The widefield images should be scaled 16 times to match reconstructed images.

### Deep-learning network training [TIMING 0.5–2 d]

21 An Ubuntu workstation with Nvidia GPU is recommended for model training. To train a personalized U-Net model, it is imperative to prepare a dataset encompassing sufficient data. This dataset consists of sparsely localized DNA-PAINT images, optional widefield images, and their paired ground truth images rendered from abundant single-molecule fluorescence signals. All image pairs are 16-bit gray-scale images and are cut into the same square size, typically 256 × 256 or 512 × 512 pixels, according to computing power and GPU memory size. Choose 5% of image pairs for the testing dataset, and the rest for the training dataset.

**[CRITICAL STEP]** The sparse localized images, widefield images and paired ground truth images are supposed to be in one-to-one correspondence at the pixel level. Any deviation needs to be corrected.

**[CRITICAL STEP]** The training dataset needs to include at least 200 pairs of cropped images with resolutions of 256 × 256 or 512 × 512 pixels, and it is ideal to have more than 1000 pairs.

**[CRITICAL STEP]** Our R script “Roiselectcut.R” can be used to crop images into small square pieces automatically: “Rscript /path/to/our/script/Roiselectcut.R /path/to/prepared/image/pair /path/to/cropped/image/dataset target-image-size”. Set “target-image-size” to 256 or 512. However, visual inspection is needed to remove the cropped images that are omitting noticeable subcellular structures.

22 For microtubule dataset preparation, simulated data can be produced and used with our scripts: “Rscript /path/to/our/script/mt-simulate.R structure-dense sparsity dataset-size /target/path/for/simulated/images”. “Structure-dense” is a positive integer describing the average microtubule number of each image, sparsity is the ratio of localization data in sparsely localized images to that in ground truth images and dataset-size is the number of simulated microtubule images. Our simulation script produces images with a resolution of 256 × 256 pixels, including the ground-truth.tif file, the widefield.tif file, and the sparsely-localized.tif file, which correspond to super-resolution images of simulated structures, structure images under diffraction-limited conditions and sparsely localized images.

23 Modify model training Python script “train.py” or “trainWF.py” (optional widefield images required) and provide training dataset path “train_data_path0”, pre-trained model path “model_load”, number of training epochs “epoch_num” (typically between 200 and 2,000) and path for model saving “model_save”. Run the script.

24 To evaluate the performance of models, a testing dataset is needed. Use sparsely localized images in the testing dataset as model input and compare the quantitative parameters calculated from the output and ground truth images. The peak signal-to-noise ratio (PSNR), root-mean-square error (RMSE), and structural similarity image measurement (SSIM) can be applied for the quantification of trained models. Run the script “performance.py” using the terminal command: “python3 /path/to/our/script/performance.py /path/to/ground-truth/image /path/to/output/image”. The PSNR, RMSE and SSIM measurements will appear in the console. The *PSNR*, *RMSE* and *SSIM* are calculated using the following functions, where *OP* and *GT* refer to the output and ground truth of testing datasets, *cov*(*GT, OP*) refers to the covariance of *GT* and *OP*, *sd* function represents standard deviation, and *c*_1_ and *c*_2_ are small positive constants.

$\quad\;\; \begin{aligned} &
RMSE=\sqrt{\mathrm{m}\mathrm{e}\mathrm{a}\mathrm{n}({(GT-OP)}^{2})}\;,\\& 
PSNR=\left\{
\begin{aligned} &
100,\;{\mathrm{when}}\;RMSE=0\\& 20lg\left(\frac{255}{RMSE}\right),\;{\mathrm{when}}\;RMSE > 0
\end{aligned}\right.\;,\\& SSIM=\{[2\mathrm{m}\mathrm{e}\mathrm{a}\mathrm{n}(GT)\mathrm{m}\mathrm{e}\mathrm{a}\mathrm{n}(OP) + {c}_{1}]\;\times\\&\qquad\quad\;
[cov(GT,OP) + {c}_{2}]\}/\\&\qquad\quad\;
\{[{\mathrm{m}\mathrm{e}\mathrm{a}\mathrm{n}}^{2}(GT) + {\mathrm{m}\mathrm{e}\mathrm{a}\mathrm{n}}^{2}(OP) + {c}_{1}]\;\times \\&\qquad\quad\;
[{sd}^{2}(GT) + {sd}^{2}(OP) + {c}_{2}]\}\;.
\end{aligned} $

**[TIMING]**

Steps 1–9, DNA-PAINT sample preparation: 2 d

Steps 10–14, DNA-PAINT raw data acquisition (TIRFM imaging): ~2 h

Steps 15–20, super-resolution image reconstruction: ~2 h

Steps 21–24, personalized U-Net model training (optional): 0.5–2 d

## ANTICIPATED RESULTS

**Figure 3:**
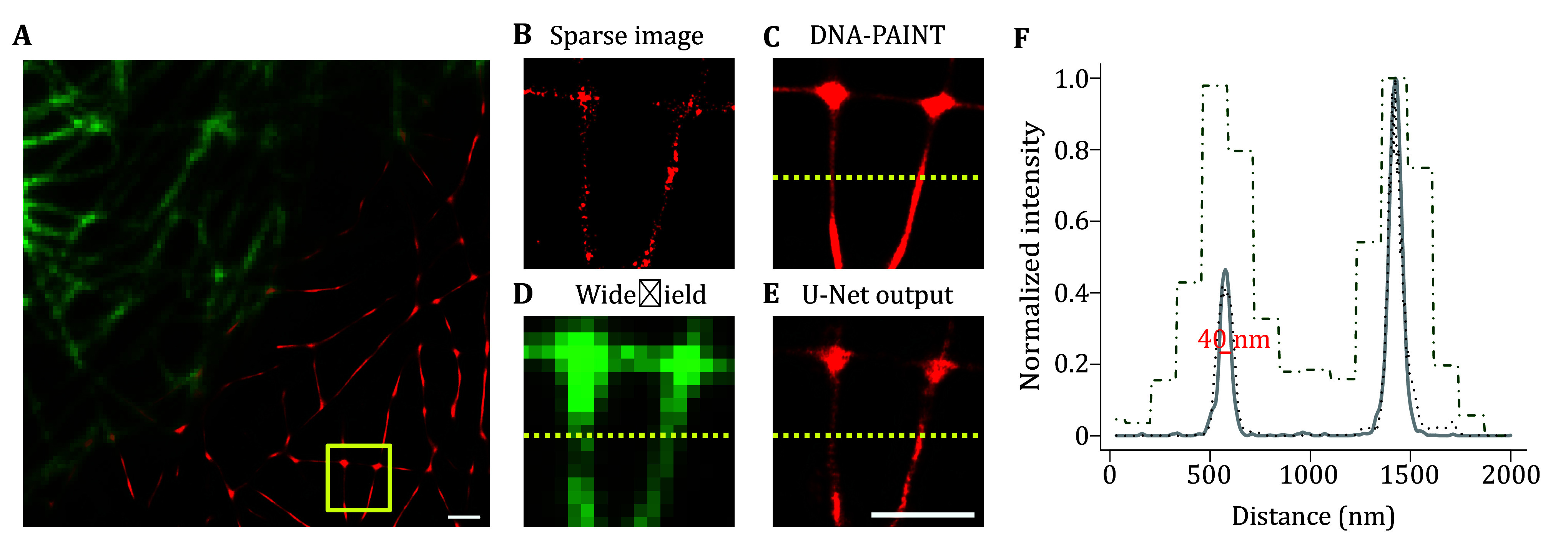
The performance of deep learning-based DNA-PAINT reconstruction. **A** Overlay of a widefield microtubule image (top left) with its DNA-PAINT reconstruction image (bottom right). Scale bar: 1 μm. **B** Sparse localization image of boxed region from Panel A with only 10% of raw data (3000 frames) used for reconstruction. **C** Conventional DNA-PAINT reconstruction (30,000 frames) result of the boxed region from Panel A. **D** Widefield image of the boxed region from Panel A. **E** Output of pre-trained U-Net model with Panels B and D as inputs. Scale bar: 1 μm. **F** Normalized intensity plot of the regions from Panels C, D and E. Gray solid curve from conventional DNA-PAINT reconstruction (**C**); black dot-dashed curve from widefield image (**D**); black dashed curve from U-Net output (**E**)

When imaging the cell sample using a 488-nm laser, it is expected that the widefield view of the target structures labeled with Alexa Fluor 488 conjugated streptavidin, such as microtubules, would be easily detectable. However, when employing TIRF microscopy with a 561-nm laser, no signal is initially detected unless the imaging buffer with Cy3B is added. Upon the addition of the imaging buffer, discrete fluorescent signals resembling points emerge and correspond to the positions of structures observed with the 488-nm laser. These blinking single molecules exhibit a continuous pattern of disappearance and reappearance. The resolution of the reconstructed images is significantly improved by PAINT (Fig. 3A), enabling visualization at a scale of 40 nm or less (Fig. 3F). Typically, widefield images encompass a larger scope of diffraction-limited structures that are out of focus, which results in misalignment between DNA-PAINT images and widefield images. In addition, by using our developed U-Net model and the widefield images generated through the simplified DNA-PAINT labeling method, the reconstructed sparse localized images can be restored, which resemble the results generated by conventional DNA-PAINT imaging but with over ten times more raw data (Figs. 3C and 3E).

## Conflict of interest

Min Zhu, Luhao Zhang, Luhong Jin, Yunyue Chen, Haixu Yang, Baohua Ji and Yingke Xu declare that they have no conflict of interest.

## References

[bAlvelid2022] (2022). Event-triggered STED imaging. Nat Methods.

[bBetzig2006] (2006). Imaging intracellular fluorescent proteins at nanometer resolution. Science.

[bChen2022] (2022). Resolution doubling in light-sheet microscopy via oblique plane structured illumination. Nat Methods.

[bChung2022] (2022). Fluorogenic DNA-PAINT for faster, low-background super-resolution imaging. Nat Methods.

[bDai2016] (2016). Optical imaging of individual biomolecules in densely packed clusters. Nat Nanotechnol.

[bFilius2020] (2020). High-speed super-resolution imaging using protein-assisted DNA-PAINT. Nano Lett.

[bGiannone2010] (2010). Dynamic superresolution imaging of endogenous proteins on living cells at ultra-high density. Biophys J.

[bGustafsson2005] (2005). Nonlinear structured-illumination microscopy: wide-field fluorescence imaging with theoretically unlimited resolution. Proc Natl Acad Sci USA.

[bHell1994] (1994). Breaking the diffraction resolution limit by stimulated emission: stimulated-emission-depletion fluorescence microscopy. Opt Lett.

[bIinuma2014] (2014). Polyhedra self-assembled from DNA tripods and characterized with 3D DNA-PAINT. Science.

[bJin2020] (2020). Deep learning enables structured illumination microscopy with low light levels and enhanced speed. Nat Commun.

[bJungmann2016] (2016). Quantitative super-resolution imaging with qPAINT. Nat Methods.

[bJungmann2014] (2014). Multiplexed 3D cellular super-resolution imaging with DNA-PAINT and Exchange-PAINT. Nat Methods.

[bJungmann2010] (2010). Single-molecule kinetics and super-resolution microscopy by fluorescence imaging of transient binding on DNA origami. Nano Lett.

[bMarx2019] (2019). Machine learning, practically speaking. Nat Methods.

[bNehme2018] (2018). Deep-STORM: super-resolution single-molecule microscopy by deep learning. Optica.

[bOleksiievets2022] (2022). Fluorescence lifetime DNA-PAINT for multiplexed super-resolution imaging of cells. Commun Biol.

[bOuyang2018] (2018). Deep learning massively accelerates super-resolution localization microscopy. Nat Biotechnol.

[bRonneberger2015] Ronneberger O, Fischer P, Brox T (2015) U-Net: convolutional networks for biomedical image segmentation. Proceedings of the Medical Image Computing and Computer-assisted Intervention – MICCAI 2015. 9351

[bRust2006] (2006). Sub-diffraction-limit imaging by stochastic optical reconstruction microscopy (STORM). Nat Methods.

[bSchindelin2012] (2012). Fiji: an open-source platform for biological-image analysis. Nat Methods.

[bSchnitzbauer2017] (2017). Super-resolution microscopy with DNA-PAINT. Nat Protoc.

[bSchueder2019] (2019). An order of magnitude faster DNA-PAINT imaging by optimized sequence design and buffer conditions. Nat Methods.

[bSharonov2006] (2006). Wide-field subdiffraction imaging by accumulated binding of diffusing probes. Proc Natl Acad Sci USA.

[bTholen2023] (2023). Beyond DNA: new probes for PAINT super-resolution microscopy. Chem Commun (Camb).

[bXie2020] (2020). 3D ATAC-PALM: super-resolution imaging of the accessible genome. Nat Methods.

[bXu2020] (2020). Super-resolution imaging reveals the evolution of higher-order chromatin folding in early carcinogenesis. Nat Commun.

[bZhu2022] (2022). DNA-PAINT imaging accelerated by machine learning. Front Chem.

